# Notes and News

**Published:** 1901-07-20

**Authors:** 


### NOTES AND NEWS.

The King conferred the honour of knighthood of the Victorian Order upon the following members of Marlborough House on the 16th inst.:—Sir Thomas Smith, Bart., F.R.C.S., Deputy Surgeon-General Henry Julius Blanc, Mr. William Henry Bennett, F.R.C.S., Mr. Thomas Hanbury. The following gentlemen were afterwards introduced and invested by his Majesty with the insignia of Commanders of the Order:—Mr. Donald William Hood, M.D., Mr. John Hammond Morgan, F.R.C.S., Mr. Charles Arthur Morris, F.R.C.S.

It appears that German medical men are going to follow the example of France and organise medical tours to the various national watering places.

The New Dental Hospital of London, Leicester Square, has received a legacy of £1,000 under the will of the late Miss Hannah Harvie, of Cheltenham.

A children's ward has been added to the Kensington Dispensary, which was opened at the beginning of the present month, by the Princess Louise.

Mr. Distin Maddick, the senior surgeon to the Italian Hospital, has received an intimation that the King of Italy will present him with the insignia of the Order of the Cavaliere d' Italia.

At a meeting of the Council of the Royal College of Surgeons held last week Mr. Howse was elected president, Mr. Jessop senior vice-president, and Mr. Howard Marsh junior vice-president.

The International Congress of Medicine will be held in April next year in Madrid. The King and the Queen Regent are patrons. The initial arrangements have already been made, and the work of the congress has been divided into six sections.

Mr. Thomas Arnold Rogers will distribute prizes to the students of the Dental Hospital of London at a conversazione to be held by the medical staff and lecturers on Thursday, August 1st, at 8 o'clock, in the new hospital in Leicester Square.

Some time ago it was decided that efforts should be made to establish a consumption sanatorium for the county of Worcester. For this purpose, Viscount Cobham has offered a site of 30 acres at a rental of £30 per annum at Romsley. Little Fairley, by which name the property is known, is a wood rising 750 feet above the sea-level; which is very suitable for the purpose. Birmingham is also considering a scheme to provide its tuberculous patients with a sanatorium, and possibly the two undertakings for town and city will be united. If so, the building will probably be arranged for 48 beds. As the County Council are powerless to establish and support buildings for the treatment of consumption, the necessary money will probably be raised by public subscription.

An African society has been formed out of the nucleus of the West African Society, which was established to commemorate Miss Mary Kingsley's work in that part of the continent. Besides a study of all matters of historic, geographic, and social interest, the society will concern itself much with matters relating to health in Africa and with the study of special forms of local disease. Already a large number of distinguished medical men have enrolled their names. The secretary is Mr. R. Sewell, and the office is at 22 Albemarle Street.

Lord James of Hereford presented the prizes to the students of Guy's Hospital Medical School last week. A large number of distinguished guests were present on the occasion, and were assembled in the new theatre in the Medical School. Lord James of Hereford gave an address, to which Mr. Cosmo Bonsor, the treasurer, replied in a vote of thanks to Lord James. He pointed out that Guy's had always been in the forefront when the call for men came. He said it might without boasting be claimed that in the history of the medical service of the South African war Guy's had played a very distinguished part. The splendid quality and extent of the work of the Imperial Yeomanry Hospital at Deelfontein had been universally acclaimed, and that hospital was in the main organised, equipped, staffed, and nursed by Guy's people under Dr. Washburn, Mr. Fripp, and Mr. Newland-Pedley. He did not forget to pay a tribute to the services rendered by Dr. Wordsworth Poole, principal medical officer to the British Legation during the siege of Pekin; and further he stated that the Government had recognised the worth of Guy's staff by the appointment of two of its members—Dr. Perry and Mr. Fripp—to act on the Army Medical Re-organisation Committee, which had now entered upon its important duties. Mr. Cosmo Bonsor pointed out that the hospital had to look to the public for £25,000 per annum for maintenance, and in addition the authorities had to face an expenditure of £180,000 on works which the age of Guy's Hospital had rendered indispensable. Many of the guests inspected the hospital after the ceremony, and much admiration was expressed at the appearance of the wards and the arrangements generally.

